# Impacts of Frailty on Prognosis in Lung Cancer Patients: A Systematic Review and Meta-Analysis

**DOI:** 10.3389/fmed.2021.715513

**Published:** 2021-07-22

**Authors:** Shuiping Dai, Ming Yang, Juan Song, Sisi Dai, Jinhui Wu

**Affiliations:** National Clinical Research Center for Geriatrics, The Center of Gerontology and Geriatrics, West China Hospital, Sichuan University, Chengdu, China

**Keywords:** frailty, lung cancer, mortality, therapeutic toxicity, prefrailty

## Abstract

**Background:** Frailty is a common geriatric syndrome and is described as a limited ability to compensate and recover from stressors. Lung cancer is largely diagnosed in old age, when frailty is common and might have predictive value on prognosis. Therefore, we performed a systematic review to evaluate the prognostic role of frailty in lung cancer.

**Methods:** The online PubMed, Web of Science, CNKI and Wanfang literature databases were searched to identify all related articles that reported the predictive value of frailty for mortality and therapeutic toxicity. Review Manager 5.3 was used to analyze results by standard meta-analysis methodology.

**Results:** Seven studies were included in this review, and only six studies with 2,359 patients were enrolled in meta-analysis. Patients in two studies received chemotherapy, two studies radiotherapy, two studies surgery, one study not reported. Compared to non-frail patients, frail patients had a higher risk of overall mortality [Hazard Ratio (HR) = 1.57, 95% confidence interval (CI), 1.32–1.87], and therapeutic toxicity [Odds Ratio (OR) = 2.60, 95% CI, 0.82–8.24]. Prefrail patients also showed higher overall mortality and therapeutic toxicity than non-frail patients (HR = 1.20, 95% CI, 1.05–1.38; OR = 1.72, 95% CI, 1.18–2.51, respectively).

**Conclusions:** Frailty is a powerful predictor of overall mortality and therapeutic toxicity in lung cancer patients.

## Introduction

Frailty is a common geriatric syndrome and is a state of age-related decline in biological reserve, decreased ability to maintain physiological balance and increased vulnerability to adverse health events (1, 2). Prefrailty is defined as a clinically silent process that predisposes individuals to frailty, and is an intermediate state between frailty and absence of frailty (3). Although the prevalence of frailty varies by the definition used, it increases with aging (4). Frailty can occur at any age, especially in those with chronic illnesses, but it is more prevalent in older adults (5). 17.4% of community-dwelling adults ages 60 years and older present with frailty, 49.3% with prefrailty, while that incidence increases to 29.5 and 59.3% respectively in those ages 85 years and older (6). The prevalence of frailty in patients with cancer is especially high (7), and it has been reported that more than half of older patients with cancer are frail at cancer diagnosis, which may increase the risk of chemotherapy intolerance, postoperative complications, and mortality (8).

Lung cancer is the leading cause of malignancy death, and causes almost one-quarter of all cancer deaths (9). Approximately 30–40% of lung cancers are diagnosed in patients aged 70 or more (10). Furthermore, the probability of developing invasive lung cancer increases with age, 0.6% among 50–59 years old, 1.8% among 60–69, 6.0% among over 70 (9). There are multiple treatment options in lung cancer, including surgery, chemotherapy, radiotherapy, etc. The prognosis of treatment is not only related to the cancer, but also related to the patient's physical and functional status. Comprehensive geriatric assessment (CGA) may help clinicians to define patients as fit, vulnerable or unfit to treatment (11, 12). Nevertheless, a phase III trail found the use of CGA to choose treatment did not improve survival but reduce treatment toxicity (13). Some studies found that frailty was associated with reduced overall survival in lung cancer patients (14, 15), and frailty assessment might inform toxicity risk (16). However, frailty was found to have no significant predictive value in postoperative outcomes (17).

In a word, frailty as a common geriatric syndrome has been tried to be used in the medical decision-making of lung cancer patients, while the predictive power and usefulness have not yet been clearly proven. Therefore, we conducted a systematic review to summarize the prognostic value of frailty in patients with lung cancer.

## Materials and Methods

### Search Strategy

We conducted a systematic literature search on the online PubMed, Web of Science, CNKI and Wanfang literature databases from the start of each database up to 12 March 2021. For PubMed, the search items included: (“lung neoplasms”[MeSH Terms] OR (“lung” AND “neoplasms”) OR (“lung” AND “cancer”) OR “lung cancer”) AND (“frailty”[MeSH Terms] OR “frailt” OR “frail”) AND (“mortality”[MeSH Terms] OR “mortality” OR “mortalities” OR (“prognosis”[MeSH Terms] OR “prognosis” OR “prognoses”) OR [(“adverse” OR “adversely” OR “adverses”) AND (“event” OR “events”)] OR (“adverse effects”) OR (“toxic” OR “toxicities” OR “toxicity”)). For Web of Science, CNKI, and Wanfang, the same MeSH-terms were used in the search strategy. The reference lists of all included studies were also consulted to locate additional references of interest. This study was conducted in accordance with Preferred Reporting Items for Systematic Reviews and Meta-analyses (PRISMA) (18) guidelines and has been registered in PROSPERO (registration number: CRD42021244311).

### Inclusion and Exclusion Criteria

All eligible literatures had to meet the following criteria: (1) used a standardized index to assess frailty in clinically diagnosed lung cancer patients; (2) reported frailty association with at least one of the following outcomes: treatment adverse effects or complications, mortality; (3) the examined associations were reported by odds ratios (OR) or hazard ratios (HR) and 95% confidence intervals (CI), or these data could be calculated; (4) the full-text was published in Chinese or English. The exclusion criteria were: (1) studies on cancer in general without specific results for lung cancer; (2) meeting, abstracts, letters, reviews, editorials and case reports; (3) duplicate publications with same sample. Two authors (SPS and SSD) screened the titles and abstracts independently to select possible eligible articles and any inclusion discrepancies were resolved through discussion.

### Data Extraction and Quality Assessment

The basic information of included studies was extracted by two reviewers (SPD and JS) including the following data: the name of the first author, publication year, country, follow-up time, sample size, frailty assessment scale, treatment methods, OR, HR, 95% CI. The extracted data of both authors were compared with one another, and any discrepancies were resolved by checking the original articles through a third reviewer (SSD).

The Newcastle-Ottawa Scale for cohort studies was used to assess the quality and risk of bias of the included studies (19). This scale contained three section: Selection, Comparability, and Outcome. A study was awarded a maximum of one point for each item within the Selection and Outcome categories, and a maximum of two points for Comparability, and a total of eight points might be achieved. The included literatures with score ≥ 6 were regarded as high-quality literatures, and those with score <6 were regarded as low-quality literatures. Two reviewers (JS and MY) independently assessed the included studies. If there was a discrepancy, all group members will discuss and solve the disagreements together.

### Statistical Analyses

Review Manager 5.3 was used to perform this meta-analysis. The heterogeneity between studies was analyzed by *I*^2^ test, which *I*^2^ > 50% indicating that a potential heterogeneity existed. The pooled HR for overall mortality, OR for treatment adverse effects or complications, and corresponding 95% CI were calculated in fixed or random effects models. If substantial heterogeneity existed, the random-effects model was employed; otherwise, the fixed-effect model was applied. The potential publication bias was estimated by visually funnel plots. A two-sided *P*-value ≤ 0.05 was considered statistically significant.

## Results

### Study Characteristics and Quality

**Figure 1:**
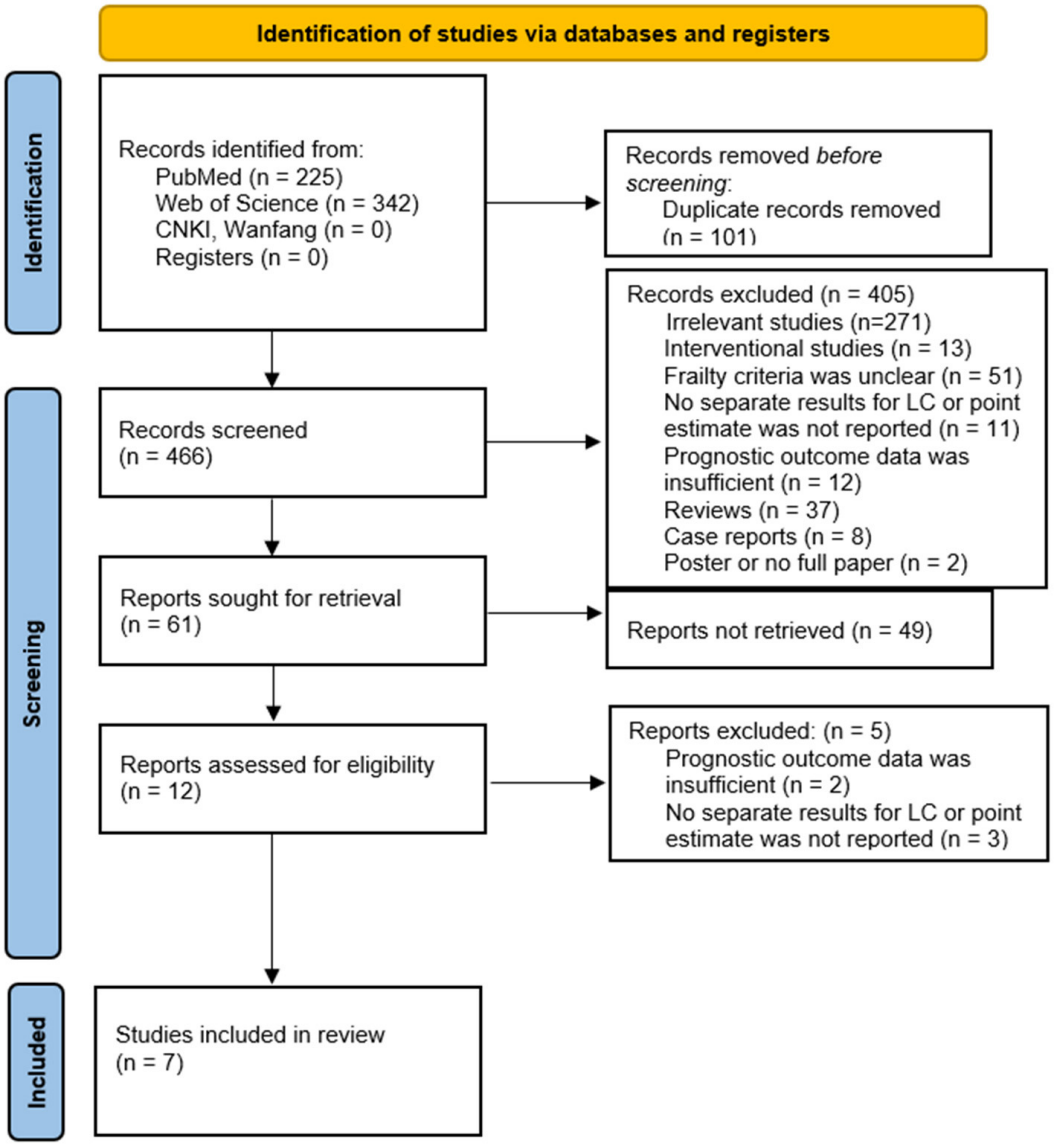
The PRISMA diagram for study selection. PRISMA: preferred reporting items for systematic reviews and meta-analyses. From (20).

As shown in Figure 1, a total of 567 articles were initially identified from the online database, of which 101 after duplication were excluded. After screening the titles and abstracts of remaining 466 records, 454 reports were excluded. 12 articles were assessed for eligibility by reading the full text, of which 2 reports excluded because of insufficient prognostic outcome data and 3 because of no separate results for lung cancer. Finally, 7 studies were included in this review (14–17, 21–23), and their characteristics were presented in Table 1. All of these studies were hospital-based registry cohort studies. A total of 3,921 lung cancer patients involved in these seven studies received different treatments, surgery in two (17, 21), radiotherapy in two (14, 15), chemotherapy in two (22), unreported in one. Three of these included studies classified patients as prefrail and frail, and one classified patients as mild, moderate and severe frailty. The assessment criteria for frailty were shown in Supplementary Table 1. All enrolled articles achieved a relatively high score equal or larger than 6 when evaluated by Newcastle-Ottawa Scale for cohort studies, and were regarded as high-quality papers. The assessment criteria of frailty used in different studies, showed in Supplementary Table 2. The largest paper (21) selected was not included in the meta-analysis because the frailty assessment measure was a cumulative risk factors score, including comorbidities and surgical factors, and had a loose conceptual relationship with frailty. Six other studies with 2,359 patients were included in meta-analysis.

**Table 1:** Baseline characteristics of the included studies.

**Study**	**Year**	**Country**	**Recruitment**	**Follow up**		**Sample size**	**Age (years)**		**Frailty**		**Females**	**Stage**	**Treatment**		**Therapeutic toxicity**		**Overall mortality**
				**Length**	**Median**	**number**	**Range**	**Median or mean**	**Scale**	**Cut off (numbers)**	**(%)**					**OR (95% CI)**	**HR (95% CI)**
Kaneda	2021	Japan	2016–2019	NR	NR	193	NR	70.4	Frailty phenotypic model	Non-frail (152), prefrail (28), frail (13)	37	1–3	Surgery	Prefrail	Postoperative complications	0.89 (0.28–2.79)	
														Frail		0.44 (0.055–3.58)	
Cespedes Feliciano	2020	America	1993–1998	19.9 years	5.8 years	822	50–79	63	Fried frailty phenotype	Non-frail (405), prefrail (239), frail (178)	100	NR	NR	Prefrail			1.09 (0.89–1.34)
														Frail			1.33 (1.05–1.68)
Wang	2019	China	2010–2017	8 years	3.9 years	1,020	≥60	65	FI-LAB	Non-frail (701), prefrail (269), frail (50)	28.6	1–4	Chemotherapy	Prefrail	Any adverse reactions	1.86 (1.25–2.77)	1.30 (1.08–1.57)
														Frail		3.48 (1.77–6.87)	2.13 (1.51–3.00)
Ruiz	2019	America	2010–2014	NR	NR	45	42–86	69	Fried Frailty Index	Frail (12), non-frail (23)	20.8	4	Chemotherapy	Frail	Treatment-related grade 3-5 toxicity	5.82 (1.06–31.81)	1.03 (0.51–2.11)
Raghavan	2018	Canada	2009–2014	76.1 months	38.8 months	140	NR	NR	Modified frailty index	Non-frail (91), frail (49)	63.9	1	Stereotactic body radiotherapy	Frail			1.98 (1.02–3.85)
Franco	2018	America	2009–2014	74.1 months	38.5 months	139	NR	74	Modified frailty index	Non-frail (101), frail (38)	51.8	1–2	Stereotactic body radiotherapy	frail			2.25 (1.44–4.44)
De la Garrza Ramos	2016	Canada	2002–2011	NR	NR	1,562	NR	62	Metastatic Spinal Tumor Frailty Index	Non-frail, mild frail, moderate frail, severe frail	NR	NR	Spine surgery	Mild frail	Complication	1.63 (0.93–2.89)	
														Moderate frail		3.52 (2.02–6.16)	
														Severe frail		5.27 (3.04–9.12)	

*NR, not reported; FI-LAB, frailty index based on laboratory variable*.

### Overall Mortality

**Figure 2:**
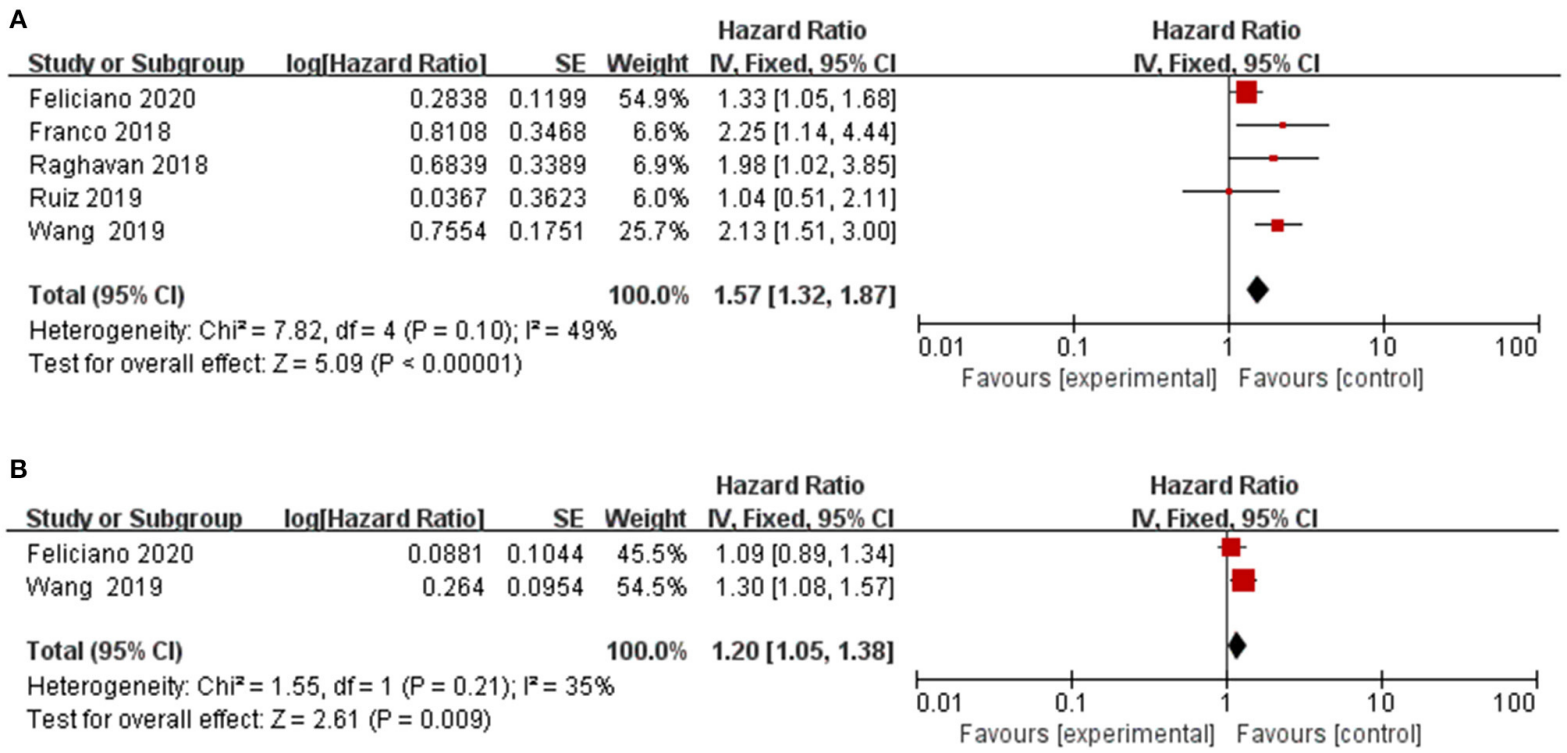
Forest plot of the association between frailty and overall mortality. **(A)** frailty and overall mortality; **(B)** prefrailty and overall mortality.

**Figure 3:**
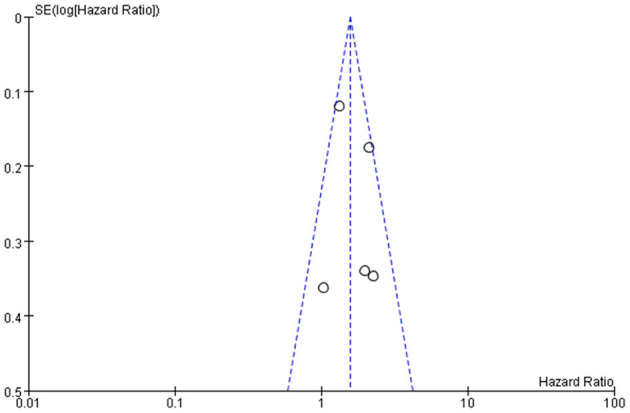
Funnel plot of frailty and overall mortality.

Frailty in association with overall mortality risk was investigated in 5 cohort studies (14–16, 22, 23). The pooled HR from the combination of included studies was 1.57 (95%CI: 1.32–1.87), which demonstrated that compared to non-frail patients, frail patients had a higher risk of overall mortality. No significant heterogeneity was observed across these pooled studies (Chi^2^ = 7.82, df = 4, *I*^2^ = 49%, *P* = 0.10) (Figure 2A). The funnel plot indicated no publication bias (Figure 3).

Two included studies explored the role of prefrailty in predicting the risks of overall mortality in patients with lung cancer (22, 23). Figure 2B showed forest plots, which showed the HR of the association between prefrailty and overall mortality. The pooled data indicated an association between prefrailty and higher overall mortality (HR = 1.20, 95% CI: 1.05–1.38). No significant heterogeneity was observed across these pooled studies (Chi^2^ = 1.55, df = 1, *I*^2^ = 35%, *P* = 0.21).

### Therapeutic Toxicity

**Figure 4:**
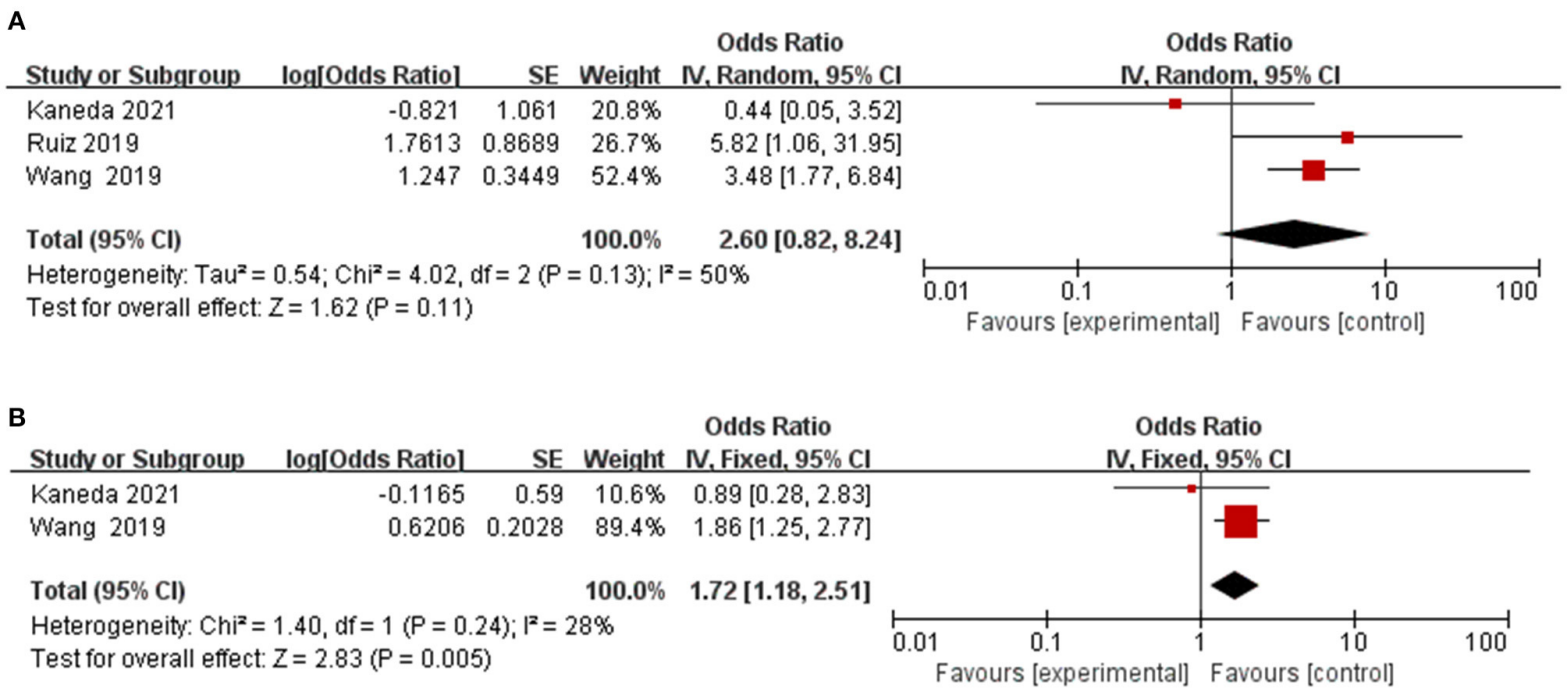
Forest plot of the association between frailty and therapeutic toxicity. **(A)** frailty and therapeutic toxicity; **(B)** prefrailty and therapeutic toxicity.

Three articles assessed the role of frailty in predicting treatment toxicity, including postoperative complications, chemotherapy adverse reactions, chemotherapy toxicity (16, 17, 22). As shown in Figure 4A, the pooled OR was 2.60 (95% CI: 0.82–8.24), indicating frailty associated with higher odds of treatment toxicity. No significant heterogeneity was observed across these pooled studies (Chi^2^ = 4.02, df = 2, *I*^2^ = 50%, *P* = 0.13). From the combination of included articles, prefrailty was associated with higher odds of treatment toxicity (OR = 1.72, 95% CI: 1.18–2.51) (Figure 4B).

De la Garza Ramos et al. (21), which assessed frailty by Metastatic Spinal Tumor Frailty Index, found that compared with patients with no frailty, patients with moderate frailty (OR = 5.15; 95% CI: 2.44–10.86), and severe frailty (OR = 5.74; 95% CI: 2.69–12.24) had significantly increased odds of inpatient mortality. Similarly, patients with mild frailty (OR = 1.88; 95% CI: 1.33–2.66), moderate frailty (OR = 3.83; 95% CI: 2.71–5.41), and severe frailty (OR = 6.97; 95% CI: 4.98–9.74) had significantly increased odds of developing a major in-hospital complication.

## Discussion

Our systematic review and meta-analysis showed poor prognosis in lung cancer patients with frailty or prefrailty. Compared to non-frail patients, frail patients had a higher risk of overall mortality and treatment toxicity. Similarly, prefrailty was associated with higher overall mortality and therapeutic toxicity.

Frailty was characterized by a decline in functioning across multiple physiological systems, while there is a progressive debate about how to define this condition (24, 25). The two most commonly used frailty assessment instruments are frailty phenotype (26) and frailty index (27). A previous review revealed that frailty prevalence greatly varies across studies, from 4 to 59%, because of lack of standardization of concepts or measures (28). Although the frailty assessment criteria were different among the studies included in this review, no significant heterogeneity was observed across these pooled studies. However, when metastatic spinal tumor frailty index (MSTFI) used in spinal patients, severely frail patients had significantly increased odds of developing a major in-hospital complication, and OR was 5.27 (95% CI, 3.04–9.12), which was higher than the pooled OR (2.60, 95% CI, 0.82–8.24) in our review. The components of MSTFI included comorbidities (e.g., anemia, chronic lung disease, and coagulopathy et al.), emergent/urgent case, and anterior or combined surgical approach, which was largely different from estimates components in other studies. At present, there are few studies comparing the predictive value of different frailty assessment instruments for prognosis. Therefore, more studies are needed in the future to explore the frailty assessment criteria suitable for different populations.

The studies included in this meta-analysis involved different therapeutic methods, including chemotherapy, radiotherapy, and surgery. Except one study found that frailty was not a significant predictor of postoperative outcomes (17), others revealed frailty was associated with overall mortality or therapeutic toxicity. Due to the small number of studies, we did not perform subgroup analyses for treatment methods. However, there may be potential differences in the predictive value of frailty for the prognosis of different treatments, which needs to be confirmed by future studies. The appearance of EGFR tyrosine kinase inhibitor and immune-checkpoint inhibitors has dramatically improved the prognosis of patients with non-small cell lung cancer, while frailty assessment is deficiency in these patients (29, 30), and prospective randomized trials addressing this question are warranted.

The patients included in this study included patients with different lung cancer stages, from stage 1 to stage 4. However, due to the limited number of studies, we did not conduct subgroup analysis on disease stages. From the early stage to the late stage of lung cancer, the proportion of frailty increased (22). However, there was no significant difference in the incidence of frailty between stage 1A and 1B/2A patients (14). Franco et al. (14) found that overall stage did not influence the survival of patients by univariate analysis (1B/2A vs. 1A, *P* = 0.46). A previous study confirmed that no significant interactions between frailty and disease stage in the recurrence-free survival (*P*_interaction_= 0.98) and OS (*P*_interaction_= 0.96) among colorectal cancer patients (31). The interactions between frailty and disease stage in the survival of lung cancer patients needs further studies to confirm.

Our review suggested that frailty was associated with prognosis of lung cancer, which was in accordance with those of studies conducted in patients with other type of cancer (7, 8). However, based on one study included in our meta-analysis, frailty seemed not to be associated with postoperative complications in lung cancer patients, which was not consistent with relevant results of studies on other types of cancer. These previous studies found that frailty was associated with increased risk of postoperative complications in patients with gastric cancer (32), pancreatic cancer (33), head and neck cancer (34), colorectal cancer (35), gynecologic cancer (36). The modified Frailty Index has been validated in several studies as a reliable measure of mortality in vascular (37), orthopedic (38), gynecologic (39), and general surgeries (40). Therefore, further studies are required to determine the association between frailty and surgery-related outcomes in lung cancer.

Many factors might affect the findings of the assessment of the impact of frailty on the prognosis of lung cancer. Studies found that unadjusted association estimates might overestimate the impacts of frailty on prognosis of lung cancer. Frailty was significantly associated with shorter OS on univariate analysis [HR = 2.22 (95% CI, 1.22–4.05)], while the value of HR decreased to 1.98 (95% CI, 1.02–3.85) (15). When adjusted for other factors, such as clinical stage and age et al., the value of HR decreased from 3.00 (95% CI, 2.15–4.18) to 2.13 (95% CI, 1.51–3.00) (22). When the factors associated with the prognosis were selected to constitute the frailty index components, the prognostic value of the frailty itself might be overestimated (21). Several studies included in this meta-analysis were derived from unadjusted association estimates and the pooled results confirmed the prognostic value of frailty. A previous study found that *I*^2^ had a substantial bias when the number of studies was small (41). Although no significant heterogeneity was observed across the pooled studies in our meta-analysis, more future studies are needed to confirm the adjusted HR of frailty because of the limited number of studied include in our review.

This meta-analysis has some limitations. First, only a small number of studies are included in this review. Therefore, we did not perform the subgroup analyses according to the different treatments. Second, the frailty assessment instruments varied across studies, and we did not perform the subgroup analyses due to limited number of studies of each assessment instruments. Third, it should be acknowledged that the number of available studies for prefrailty was small for robust conclusions to be drawn. Further studies are needed to establish the strength of association between prefrailty and lung cancer prognosis.

## Conclusions

Frailty and prefrailty seem to have a significant impact on the mortality and therapeutic toxicity of patients with lung cancer. Therefore, frailty assessment is important before treatment, which may affection treatment decisions. More prospective studies are needed to explore the prognostic value of frailty in lung cancer patients receiving different treatments.

## Data Availability Statement

The original contributions presented in the study are included in the article/Supplementary Material, further inquiries can be directed to the corresponding author/s.

## Author Contributions

ShD, MY, and JW: conception or design and drafting the work or revising. ShD, JS, and SiD: acquisition, analysis, or interpretation of data. All authors contributed to the article and approved the submitted version.

## Conflict of Interest

The authors declare that the research was conducted in the absence of any commercial or financial relationships that could be construed as a potential conflict of interest.
