# Supplementary material for: Impacts of Frailty on Prognosis in Lung Cancer Patients: A Systematic Review and Meta-Analysis
Source: Front Med (Lausanne). 2021 Jul 22;8:715513. doi: 10.3389/fmed.2021.715513 (PMC8339469; doi:10.3389/fmed.2021.715513)
Supplement: Supplementary file 1 [file Table_1.DOC]

Supplementary Table 1 The quality assessment of included articles by Newcastle-Ottawa Scale for cohort studies

| Author | Year | Subject selection | | | | Comparability | | Outcome | | | Score |
| --- | --- | --- | --- | --- | --- | --- | --- | --- | --- | --- | --- |
|  |  | Representativeness of the exposed cohort | Selection of the non exposed cohort | Ascertainment of exposure | Demonstration that outcome of interest was not present at start of study | select the most important factor | study controls for any additional factor | Assessment of outcome | Was follow-up long enough for outcomes to occur | Adequacy of follow up of cohorts |  |
| Kaneda | 2021 | 0 | 0 | 1 | 1 | 1 | 0 | 1 | 1 | 1 | 6 |
| Cespedes Feliciano | 2020 | 0 | 0 | 1 | 1 | 1 | 0 | 1 | 1 | 1 | 6 |
| Wang | 2019 | 0 | 0 | 1 | 1 | 1 | 1 | 1 | 1 | 1 | 7 |
| Ruiz | 2019 | 0 | 0 | 1 | 1 | 1 | 0 | 1 | 1 | 1 | 6 |
| Raghavan | 2018 | 0 | 0 | 1 | 1 | 1 | 1 | 1 | 1 | 1 | 7 |
| Franco | 2018 | 0 | 0 | 1 | 1 | 1 | 1 | 1 | 1 | 1 | 7 |
| De la Garrza Ramos | 2016 | 0 | 0 | 1 | 1 | 1 | 0 | 1 | 1 | 1 | 6 |

Supplementary Table 2 The assessment criteria of frailty of included articles

| Author | Year | Scale | Components | Score range | Cut off | | | |
| --- | --- | --- | --- | --- | --- | --- | --- | --- |
|  |  |  |  |  | Frail |  | Prefrail | Non-frail |
| Kaneda | 2021 | Frailty phenotypic model | unintentional weight loss, fatigue, inactivity, poor grip strength, and slow walking speed | 0-5 | 3-5 |  | 1-2 | 0 |
| Cespedes Feliciano | 2020 | Fried frailty phenotype | weight loss of at least 15 lb, low physical activity, muscle weakness or impaired walking and exhaustion | 0-5 | 3-5 |  | 1-2 | 0 |
| Wang | 2019 | FI-LAB | 44 laboratory variables | 0-1 | ≥0.35 |  | 0.2-0.35 | <0.2 |
| Ruiz | 2019 | Fried Frailty Index | unintentional weight loss, exhaustion, low self-reported physical activity, slow usual gait speed, low grip strength | 0-5 | 3-5 |  | 1-2 | 0 |
| Raghavan | 2018 | Modified frailty index | diabetes, performance status≥2, hypertension requiring medication, chronic obstructive pulmonary disease, coronary or cardiac disease, congestive heart failure, transient ischemic attack or cerebrovascular accident, and peripheral vascular disease | 0-8 | 3-8 |  | - | 0-2 |
| Franco | 2018 | Modified frailty index | performance status≥2, impaired sensorium, diabetes mellitus, chronic/acute lung disease, myocardial infarction in past ≤6 months, hospitalization for congestive heart failure in past≤6 months, coronary or cardiac disease, hypertension on medications, history of transient ischemic attack, cerebrovascular accident or stroke with neurological deficits, and peripheral vascular disease. | 0-11 | 2-11 |  | - | 0-1 |
|  |  |  |  |  | Severe frailty | Moderate frailty | Mild frailty | No frailty |
| De la Garrza Ramos | 2016 | Metastatic Spinal Tumor Frailty Index (MSTFI) | Anemia, Chronic lung disease, Coagulopathy, Electrolyte abnormalities, Pulmonary circulation disorders, Renal failure, Malnutrition, Emergent/urgent case, Anterior or combined surgical approach | 0-10 | 3-10 | 2 | 1 | 0 |

FI-LAB, frailty index based on laboratory variable
